# The 100 top-cited articles in diabetic kidney disease: a bibliometric analysis

**DOI:** 10.1080/0886022X.2021.1919528

**Published:** 2021-05-03

**Authors:** Zineng Huang, Huifang Zhang, Ying Luo, Cong Wei, Yuee Zhao, Ying Huang, Lei Zhang, Wei Chen, Liyu He, Hong Liu, Lin Sun, Fuyou Liu, Li Xiao

**Affiliations:** a Department of Nephrology, The Second Xiangya Hospital, Central South University, Changsha, China; b Department of Nephrology, Zhuzhou Central Hospital, Zhuzhou, China

**Keywords:** Bibliometric analysis, citation analysis, diabetic kidney disease, top cited

## Abstract

**Background:**

Tremendous scientific researches have been conducted in the field of diabetic kidney disease (DKD), while few bibliometric analyses have been performed. We aim to identify 100 top-cited published articles about DKD and analyze their main characteristics quantitatively.

**Methods:**

Web of Science was searched with the term ‘diabetic kidney disease’ OR ‘diabetic nephropathy’ to identify the top 100 most cited articles. For articles meeting the predefined criteria, the following data were extracted and analyzed: citation ranking, publication year, publication journal, journal impact factor, country and institution, authors, study type, and keywords.

**Results:**

The highest number of citations was 4753 times. The median average citations per year was 21.8 (IQR, 16.6–33.0). Most articles focused on the pathogenesis and treatment. These articles were published in 25 different journals and the *Journal of the American Society of Nephrology* published the greatest number (20%). Forty-three articles (43%) originated from the United States. The University of Groningen was the leading institute, contributing five top-cited articles. The most frequent first author was de Zeeuw (*n* = 4), followed by Parving (*n* = 3). There was no correlation between the average citations and the number of authors, the number of institutes, or the number of funds, respectively. Experimental animal study was the research type most frequently conducted (*n* = 30), followed by observational study (*n* = 24). Keyword analysis revealed transforming growth factor-β, oxidative stress, proteinuria, and renin–angiotensin–aldosterone system interruption are classic research topics. Sodium-glucose cotransporter 2 inhibitors, glucagon-like peptide 1 receptor agonists, and anti-inflammatory agents are the emerging trends of DKD.

**Conclusions:**

This bibliometric analysis helps in identifying the milestones, inadequacies, classic hotspots, and emerging trends of DKD. Pathogenesis and treatment are core themes in DKD research, while high-quality articles on the prediction and biomarker are insufficient. New analyzing metrics are needed to assess the actual impact of these top-cited articles on clinical practice.

## 1. Introduction

Diabetic kidney disease (DKD) is the leading cause of end-stage renal disease (ESRD) and is also associated with substantial cardiovascular risk and mortality [1,2]. Despite much efforts on blood pressure control, glycemic control, and lifestyle interventions, DKD is still a progressive disease. There is a boom of scientific literature in the field of DKD along with the growing DKD incidence rate worldwide. In Web of Science Core Collection, searching with ‘diabetic kidney disease’ or ‘diabetic nephropathy’ and their permutations retrieved approximately 31,373 literatures published since 1985.

Bibliometrics is a method of quantitative science to analyze research publications and increased markedly concomitant with the sharp expansion of literature. Citation analysis is an important methodology frequently used in bibliometrics and allows identification of the seminal papers, outstanding institutes or scientists, progressions, and the emerging trends in a specific field [3,4].

There are unchanging parts and changing parts in the DKD research field over the past few decades. For example, the renin–angiotensin–aldosterone system (RAAS) blockers are still first-line antihypertensive agents for DKD renoprotection. Randomized control trials indicated angiotensin-converting enzyme inhibitor (ACEI)/angiotensin receptor blockade (ARB) combination therapy or direct renin inhibition as add-on therapy to ACEI/ARB treatment is not superior, or even inferior to single RASS blocker [5–8]. Meanwhile, growing evidence is promoting the shift from classic oral hypoglycemic agents to sodium-glucose cotransporter 2 (SGLT2) inhibitors and glucagon-like peptide 1 receptor agonists (GLP-1RAs), as the latter two may offer renoprotection independent of glycemic control [9–13]. A quantitative analysis of the top-cited articles would help in revealing these milestones and progressions, identifying the present shortages and emerging trends, and guiding the future research direction of DKD. Nevertheless, no quantitative study of the top 100 most cited articles in the field of DKD has been performed.

This study was conducted to evaluate the seminal scientific output in the field of DKD through quantitatively analyzing the detailed characteristics of the selected articles, including citation ranking, publication year, publication journal, journal impact factor (IF), country and institution of origin, authorship, study type, and keywords.

## 2. Methods

### 2.1. Identification of the 100 top-cited articles

In December 2020, Thomson Reuters Web of Science [14] was used to query published articles in the field of DKD with the retrieval strategy: TS=(‘diabetic kidney disease’) OR TS=(‘diabetic nephropathy’). The inclusion criteria were as follows: publication with core subject as DKD; defined as ‘journal article’, or ‘original article’ or its synonymous expression. We excluded position papers, guidelines, reviews, meta-analysis, letters, case reports, or editorials. For revealing the classic hotspots and the emerging trends, we restricted the publication year from 2000 to 2020. The 100 top-cited articles were independently assessed and characterized by two reviewers (Z. Huang and H. Zhang). Discrepancies were resolved by consensus.

### 2.2. Citation analysis metrics

The following data of each eligible article were extracted: publication title, the number of total citations, average citations per year since publication, publication year, publication journal, journal IF, country and institution of origin, authors, type of the study, and keywords. Keywords were manually confirmed and unified before analysis (e.g., tgf-beta, TGF-beta signaling, transforming growth factor beta, transforming growth factor-β, growth-factor-beta, and so forth, are unified as TGF-beta). Keywords Plus which provided little information was eliminated from analysis (e.g., disease). The overall design of this study referred to several previous bibliometric literatures [15–18].

### 2.3. Statistical analysis and bibliometric networks

Statistical analysis was performed by the R Programming Language (version 4.0.2, Vienna, Austria) [19]. Continuous variables were expressed as the means with standard deviations (SDs), while discrete variables were expressed as the medians with interquartile ranges (IQRs). Spearman's rank correlation coefficient was used for correlation analysis. Data visualization was performed by Python package Pandas (version 1.1.0) and Matplotlib (version 3.3.1) [20,21]. VOS viewer software (version 1.6.15, Leiden University Center for Science and Technology Studies, Leiden, Netherlands) was used to create the construct networks for country coauthorship and institution coauthorship [22].

## 3. Results

### 3.1. The identified top 100 cited articles

The 100 top-cited articles were summarized in descending order according to the number of total citations (Table 1). The most frequently cited article in the field of DKD has been cited 4753 times. The median number of citations was 282 (IQR, 241–372). As total citations partially depend on publication year, we also add a column of average citations adjusted by publication year. In the list of top 10 articles according to the average citations, six articles were published in recent 10 years (data not shown). The maximum and median average citations per year were 237.7 and 21.8 (IQR, 16.6–33.0), respectively.

**Table 1.** The top 100 cited articles in diabetic kidney disease.

Citation^a^	Average citation^b^	Funds^c^	Institutes^d^	Authors^e^	First institute^f^	Country^g^	IF^h^	References
4753	237.7	1	8	10	Brigham and Women's Hospital	USA	74.7	Brenner, B.M., et al. Effects of losartan on renal and cardiovascular outcomes in patientswith type 2 diabetes and nephropathy. New England Journal of Medicine 345, 861–869(2001).
2249	112.5	2	5	6	Steno Diabetes Center	Denmark	74.7	Parving, H.H., et al. The effect of irbesartan on the development of diabetic nephropathyin patients with type 2 diabetes. New England Journal of Medicine 345, 870–878 (2001).
1035	57.5	19	2	6	University of Oxford	England	8.9	Adler AI, et al. Development and progression of nephropathy in type 2 diabetes: the United Kingdom Prospective Diabetes Study (UKPDS 64). Kidney Int. 2003;63:225–232.
775	59.6	1	6	5	Rigshospitalet	Denmark	74.7	Parving, H.H., et al. Aliskiren combined with losartan in type 2 diabetes and nephropathy.New England Journal of Medicine 358, 2433–2446 (2008).
762	42.3	2	6	264	Harvard Medical School	USA	45.5	Steffes, M.W., et al. Sustained effect of intensive treatment of type 1 diabetes mellitus ondevelopment and progression of diabetic nephropathy - The Epidemiology of DiabetesInterventions and Complications (EDIC) study. Jama-Journal of the American MedicalAssociation 290, 2159–2167 (2003).
745	49.7	7	2	4	Mount Sinai School of Medicine	USA	7.7	Susztak K, Raff AC, Schiffer M, et al. Glucose-induced reactive oxygen species cause apoptosis of podocytes and podocyte depletion at the onset of diabetic nephropathy. Diabetes. 2006;55:225–233.
716	34.1	9	2	9	University of Pennsylvania	USA	9.4	Ziyadeh FN, et al. Long-term prevention of renal insufficiency, excess matrix gene expression, and glomerular mesangial matrix expansion by treatment with monoclonal antitransforming growth factor-beta antibody in db/db diabetic mice. Proc Natl Acad Sci USA. 2000;97:8015–8020.
684	40.2	NA^i^	8	10	University of Groningen	Netherlands	8.9	de Zeeuw, D., et al. Proteinuria, a target for renoprotection in patients with type 2 diabeticnephropathy: Lessons from RENAAL. Kidney International 65, 2309–2320 (2004).
613	87.6	4	5	11	University of Toronto	Canada	23.6	Cherney, D.Z.I., et al. Renal Hemodynamic Effect of Sodium-Glucose Cotransporter 2Inhibition in Patients With Type 1 Diabetes Mellitus. Circulation 129, 587-597 (2014).
573	71.6	2	11	16	University of Pittsburgh	USA	74.7	Fried, L.F., et al. Combined Angiotensin Inhibition for the Treatment of DiabeticNephropathy. New England Journal of Medicine 369, 1892–1903 (2013).
572	30.1	2	2	4	University of Colorado	USA	8.9	Schrier RW, Estacio RO, Esler A, et al. Effects of aggressive blood pressure control in normotensive type 2 diabetic patients on albuminuria, retinopathy and strokes. Kidney Int. 2002;61:1086–1097.
571	51.9	NA	11	16	Leiden University	Netherlands	9.3	Tervaert TWC, et al. Pathologic classification of diabetic nephropathy. J Am Soc Nephrol. 2010;21:556–563.
549	32.3	11	7	10	University of Groningen	Netherlands	23.6	de Zeeuw, D., et al. Albuminuria, a therapeutic target for cardiovascular protection in type2 diabetic patients with nephropathy. Circulation 110, 921–927 (2004).
539	41.5	11	3	5	Harvard Medical School	USA	9.3	Zeisberg EM, Potenta SE, Sugimoto H, et al. Fibroblasts in kidney fibrosis emerge via endothelial-to-mesenchymal transition. J Am Soc Nephrol. 2008;19:2282–2287.
524	37.4	2	3	7	Beckman Research Institute	USA	9.4	Kato, M., et al. MicroRNA-192 in diabetic kidney glomeruli and its function in TGF-betainducedcollagen expression via inhibition of E-box repressors. Proceedings of theNational Academy of Sciences of the United States of America 104, 3432–3437 (2007).
514	51.4	5	4	6	University of Washington	USA	45.5	de Boer IH, et al. Temporal trends in the prevalence of diabetic kidney disease in the United States. JAMA. 2011;305:2532–2539.
505	50.5	1	11	12	Hannover Medical School	Germany	74.7	Haller H, et al. Olmesartan for the delay or prevention of microalbuminuria in type 2 diabetes. N Engl J Med. 2011;364:907–917.
504	28.0	4	4	6	Joslin Diabetes Center	USA	74.7	Perkins, B.A., et al. Regression of microalbuminuria in type 1 diabetes. New EnglandJournal of Medicine 348, 2285-2293 (2003).
495	61.9	1	13	20	University of Groningen	Netherlands	74.7	de Zeeuw, D., et al. Bardoxolone Methyl in Type 2 Diabetes and Stage 4 Chronic KidneyDisease. New England Journal of Medicine 369, 2492–2503 (2013).
489	44.5	1	7	11	University of Groningen	Netherlands	60.4	de Zeeuw, D., et al. Selective vitamin D receptor activation with paricalcitol for reduction of albuminuria in patients with type 2 diabetes (VITAL study): a randomised controlledtrial. Lancet 376, 1543–1551 (2010).
441	24.5	5	4	18	Columbia University	USA	3.5	Wendt TM, et al. RAGE drives the development of glomerulosclerosis and implicates podocyte activation in the pathogenesis of diabetic nephropathy. Am J Pathol. 2003;162:1123–1137.
433	36.1	2	4	12	Beckman Research Institute	USA	20.0	Kato, M., et al. TGF-beta activates Akt kinase through a microRNA-dependent amplifyingcircuit targeting PTEN. Nature Cell Biology 11, 881-U263 (2009).
393	19.7	3	5	12	Kanazawa University	Japan	11.9	Yamamoto Y, et al. Development and prevention of advanced diabetic nephropathy in RAGE-overexpressing mice. J Clin Invest. 2001;108:261–268.
389	24.3	8	2	7	University of Texas	USA	4.2	Gorin Y, et al. Nox4 NAD(P)H oxidase mediates hypertrophy and fibronectin expression in the diabetic kidney. J Biol Chem. 2005;280:39616–39626.
377	94.3	2	6	9	KfH Kidney Center	Germany	74.7	Mann, J.F.E., et al. Liraglutide and Renal Outcomes in Type 2 Diabetes. New EnglandJournal of Medicine 377, 839–848 (2017).
371	53.0	NA	14	15	University of Washington	USA	16.0	Tuttle, K.R., et al. Diabetic Kidney Disease: A Report From an ADA ConsensusConference. Diabetes Care 37, 2864-2883 (2014).
364	18.2	1	3	6	Ghent University	Belgium	9.3	De Vriese A, et al. Antibodies against vascular endothelial growth factor improve early renal dysfunction in experimental diabetes. J Am Soc Nephrol. 2001;12:993–1000.
356	17.0	3	4	12	Shiga University of Medical Science	Japan	5.0	Koya D, et al. Amelioration of accelerated diabetic mesangial expansion by treatment with a PKC beta inhibitor in diabetic db/db mice, a rodent model for type 2 diabetes. FASEB J. 2000;14:439–447.
356	29.7	9	5	11	University of Helsinki	Finland	7.7	Groop PH, et al. The presence and severity of chronic kidney disease predicts all-cause mortality in type 1 diabetes. Diabetes. 2009;58:1651–1658.
352	20.7	6	7	14	Keio University	Japan	11.9	Ichihara, A., et al. Inhibition of diabetic nephropathy by a decoy peptide corresponding tothe "handle'' region for nonproteolytic activation of prorenin. Journal of Clinical Investigation 114, 1128-1135 (2004).
350	19.4	1	9	11	University of Minnesota	USA	8.9	Keane, W.F., et al. The risk of developing end-stage renal disease in patients with type 2diabetes and nephropathy: The RENAAL Study. Kidney International 63, 1499-1507(2003).
348	16.6	NA	5	9	Columbia University	USA	9.3	Tanji N, et al. Expression of advanced glycation end products and their cellular receptor RAGE in diabetic nephropathy and nondiabetic renal disease. J Am Soc Nephrol. 2000;11:1656–1666.
347	20.4	2	1	5	Monash Medical Centre	Australia	8.9	Chow F, Ozols E, Nikolic-Paterson DJ, et al. Macrophages in mouse type 2 diabetic nephropathy: correlation with diabetic state and progressive renal injury. Kidney Int. 2004;65:116–128.
344	34.4	5	10	17	University of Michigan	USA	11.9	Inoki K, et al. mTORC1 activation in podocytes is a critical step in the development of diabetic nephropathy in mice. J Clin Invest. 2011;121:2181–2196.
335	41.9	1	8	10	Royal Victoria Hospital and McGill University	Canada	5.9	Yale, J.F., et al. Efficacy and safety of canagliflozin in subjects with type 2 diabetes andchronic kidney disease. Diabetes Obesity & Metabolism 15, 463-473 (2013).
329	18.3	1	3	4	Mito Red Cross Hospital	Japan	7.7	Sato A, Hayashi K, Naruse M, et al. Effectiveness of aldosterone blockade in patients with diabetic nephropathy. Hypertension. 2003;41:64–68.
324	32.4	10	15	23	University Hospital Freiburg	Germany	11.9	Godel M, et al. Role of mTOR in podocyte function and diabetic nephropathy in humans and mice. J Clin Invest. 2011;121:2197–2209.
317	15.9	1	3	10	University of Melbourne	Australia	11.9	Oldfield, M.D., et al. Advanced glycation end products cause epithelial-myofibroblasttransdifferentiation via the receptor for advanced glycation end products (RAGE). Journalof Clinical Investigation 108, 1853-1863 (2001).
314	16.5	4	1	5	University of Tokyo	Japan	8.9	Onozato ML, Tojo A, Goto A, et al. Oxidative stress and nitric oxide synthase in rat diabetic nephropathy: effects of ACEI and ARB. Kidney Int. 2002;61:186–194.
311	31.1	3	4	7	University of Arizona	USA	7.7	Zheng HT, et al. Therapeutic potential of Nrf2 activators in streptozotocin-induced diabetic nephropathy. Diabetes. 2011;60:3055–3066.
310	17.2	2	15	17	University of Colorado	USA	21.3	Berl T, et al. Cardiovascular outcomes in the irbesartan diabetic nephropathy trial of patients with type 2 diabetes and overt nephropathy. Ann Intern Med. 2003;138:542–549.
308	20.5	2	3	6	Monash Medical Centre	Australia	8.9	Chow FY, et al. Monocyte chemoattractant protein-1 promotes the development of diabetic renal injury in streptozotocin-treated mice. Kidney Int. 2006;69:73–80.
307	14.6	1	2	7	Henry Ford Hospital	USA	9.3	Riser BL, et al. Regulation of connective tissue growth factor activity in cultured rat mesangial cells and its expression in experimental diabetic glomerulosclerosis. J Am Soc Nephrol. 2000;11:25–38.
306	20.4	2	6	5	Steno Diabetes Center	Denmark	8.9	Parving, H.H., et al. Prevalence and risk factors for microalbuminuria in a referred cohortof type II diabetic patients: A global perspective. Kidney International 69, 2057–2063(2006).
306	18.0	1	11	13	University of Virginia Health System	USA	3.4	Bolton WK, et al. Randomized trial of an inhibitor of formation of advanced glycation end products in diabetic nephropathy. Am J Nephrol. 2004;24:32–40.
295	14.8	2	1	4	University of Minnesota	USA	8.9	Steffes, M.W., Schmidt, D., McCrery, R., Basgen, J.M. & Int Diabet Nephropathy Study,G. Glomerular cell number in normal subjects and in type 1 diabetic patients. KidneyInternational 59, 2104–2113 (2001).
293	22.5	3	1	7	The University of Chicago	USA	5.0	Wang Q, et al. MicroRNA-377 is up-regulated and can lead to increased fibronectin production in diabetic nephropathy. FASEB J. 2008;22:4126–4135.
290	18.1	12	7	8	Case Western Reserve University	USA	7.7	Genuth S, et al. Glycation and carboxymethyllysine levels in skin collagen predict the risk of future 10-year progression of diabetic retinopathy and nephropathy in the diabetes control and complications trial and epidemiology of diabetes interventions and complications participants with type 1 diabetes. Diabetes. 2005;54:3103–3111.
285	25.9	1	6	7	Schwabing General Hospital	Germany	9.3	Mann, J.F.E., et al. Avosentan for Overt Diabetic Nephropathy. Journal of the AmericanSociety of Nephrology 21, 527-535 (2010).
283	18.9	8	4	14	University of Munich	Germany	7.7	Schmid H, et al. Modular activation of nuclear factor-kappa B transcriptional programs in human diabetic nephropathy. Diabetes. 2006;55:2993–3003.
282	14.8	4	2	7	Kurume University	Japan	4.2	Yamagishi S, et al. Advanced glycation end product-induced apoptosis and overexpression of vascular endothelial growth factor and monocyte chemoattractant protein-1 in human-cultured mesangial cells. J Biol Chem. 2002;277:20309–20315.
270	19.3	5	10	18	University of Heidelberg	Germany	36.1	Isermann B, et al. Activated protein C protects against diabetic nephropathy by inhibiting endothelial and podocyte apoptosis. Nat Med. 2007;13:1349–1358.
269	22.4	7	4	14	Baker IDI Heart and Diabetes Institute	Australia	9.3	Coughlan, M.T., et al. RAGE-induced Cytosolic ROS Promote Mitochondrial SuperoxideGeneration in Diabetes. Journal of the American Society of Nephrology 20, 742-752(2009).
267	19.1	4	5	10	University of Florida	USA	9.3	Nakagawa T, et al. Diabetic endothelial nitric oxide synthase knockout mice develop advanced diabetic nephropathy. J Am Soc Nephrol. 2007;18:539–550.
266	20.5	2	3	7	Albert Einstein College of Medicine	USA	36.1	Niranjan T, et al. The Notch pathway in podocytes plays a role in the development of glomerular disease. Nat Med. 2008;14:290–298.
266	16.6	6	4	12	University of Helsinki	Finland	16.0	Thorn LM, et al. Metabolic syndrome in type 1 diabetes – association with diabetic nephropathy and glycemic control (the FinnDiane study). Diabetes Care. 2005;28:2019–2024.
264	15.5	2	2	9	Universidad Austral Bueras	Chile	4.5	Mezzano S, et al. NF-kappa B activation and overexpression of regulated genes in human diabetic nephropathy. Nephrol Dial Transplant. 2004;19:2505–2512.
263	23.9	2	1	6	Cardiff University	England	9.3	Krupa A, et al. Loss of microRNA-192 promotes fibrogenesis in diabetic nephropathy. J Am Soc Nephrol. 2010;21:438–447.
263	14.6	2	1	5	University of Essex	England	7.7	Babaei-Jadidi R, Karachalias N, Ahmed N, et al. Prevention of incipient diabetic nephropathy by high-dose thiamine and benfotiamine. Diabetes. 2003;52:2110–2120.
262	32.8	14	12	24	University of California San Diego	USA	9.3	Sharma K, et al. Metabolomics reveals signature of mitochondrial dysfunction in diabetic kidney disease. J Am Soc Nephrol. 2013;24:1901–1912.
262	26.2	3	5	6	Albert Einstein College of Medicine	USA	7.7	Woroniecka KI, et al. Transcriptome analysis of human diabetic kidney disease. Diabetes. 2011;60:2354–2369.
260	14.4	6	3	7	University of Turin	Italy	7.7	Doublier S, et al. Nephrin expression is reduced in human diabetic nephropathy – evidence for a distinct role for glycated albumin and angiotensin II. Diabetes. 2003;52:1023–1030.
258	21.5	5	3	6	University of Pittsburgh	USA	9.3	Dai CS, et al. Wnt/beta-catenin signaling promotes podocyte dysfunction and albuminuria. J Am Soc Nephrol. 2009;20:1997–2008.
256	16.0	NA	11	11	Monash Medical Centre	Australia	6.6	Atkins RC, et al. Proteinuria reduction and progression to renal failure in patients with type 2 diabetes mellitus and overt nephropathy. Am J Kidney Dis. 2005;45, 281–287.
253	18.1	4	5	7	Joslin Diabetes Center	USA	9.3	Perkins, B.A., et al. Microalbuminuria and the risk for early progressive renal functiondecline in type 1 diabetes. Journal of the American Society of Nephrology 18, 1353-1361(2007).
253	15.8	NA	3	10	Kawasaki Medical School	Japan	3.1	Satoh M, et al. NAD(P)H oxidase and uncoupled nitric oxide synthase are major sources of glomerular superoxide in rats with experimental diabetic nephropathy. Am J Physiol Renal Physiol. 2005;288:F1144–F1152.
251	12.0	5	5	15	Kanazawa University	Japan	8.9	Wada T, et al. Up-regulation of monocyte chemoattractant protein-1 in tubulointerstitial lesions of human diabetic nephropathy. Kidney Int. 2000;58:1492–1499.
251	22.8	3	2	11	University of Ottawa	Canada	3.1	Sedeek M, et al. Critical role of Nox4-based NADPH oxidase in glucose-induced oxidative stress in the kidney: implications in type 2 diabetic nephropathy. Am J Physiol Renal Physiol. 2010;299:F1348–F1358.
250	13.9	2	2	7	University of Melbourne	Australia	7.7	Tikellis, C., et al. Characterization of renal angiotensin-converting enzyme 2 in diabeticnephropathy. Hypertension 41, 392-397 (2003).
249	22.6	4	3	6	University of Arizona	USA	7.7	Jiang T, et al. The protective role of Nrf2 in streptozotocin-induced diabetic nephropathy. Diabetes. 2010;59:850–860.
249	27.7	2	5	9	Baylor College of Medicine	USA	21.6	Wang WJ, et al. Mitochondrial fission triggered by hyperglycemia is mediated by ROCK1 activation in podocytes and endothelial cells. Cell Metab. 2012;15:186–200.
249	22.6	4	8	20	University of Bristol	England	21.6	Welsh GI, et al. Insulin signaling to the glomerular podocyte is critical for normal kidney function. Cell Metab. 2010;12:329–340.
249	15.6	2	18	19	Cleveland Clinic Foundation	USA	9.3	Pohl MA, et al. Independent and additive impact of blood pressure control and angiotensin II receptor blockade on renal outcomes in the Irbesartan Diabetic Nephropathy Trial: clinical implications and limitations. J Am Soc Nephrol. 2005;16:3027–3037.
243	48.6	8	5	7	University of Washington	USA	45.5	Afkarian M, et al. Clinical manifestations of kidney disease among US adults with diabetes, 1988–2014. JAMA. 2016;316:602–610.
242	13.4	5	2	10	Kumamoto University School of Medicine	Japan	7.7	Kiritoshi S, et al. Reactive oxygen species from mitochondria induce cyclooxygenase-2 gene expression in human mesangial cells – potential role in diabetic nephropathy. Diabetes. 2003;52:2570–2577.
240	13.3	4	2	8	Steno Diabetes Center	Denmark	16.0	Hovind, P., et al. Decreasing incidence of severe diabetic microangiopathy in type 1diabetes. Diabetes Care 26, 1258-1264 (2003).
239	12.6	13	5	6	Aarhus University Hospital	Denmark	7.7	Flyvbjerg A, et al. Amelioration of long-term renal changes in obese type 2 diabetic mice by a neutralizing vascular endothelial growth factor antibody. Diabetes. 2002;51:3090–3094.
239	26.6	10	6	12	Joslin Diabetes Center	USA	9.3	Niewczas MA, et al. Circulating TNF receptors 1 and 2 predict ESRD in type 2 diabetes. J Am Soc Nephrol. 2012;23:507–515.
238	18.3	3	1	6	University of Pittsburgh	USA	3.5	Li YJ, et al. Epithelial-to-mesenchymal transition is a potential pathway leading to podocyte dysfunction and proteinuria. Am J Pathol. 2008;172:299–308.
238	23.8	4	2	11	JDRF Danielle Alberti Memorial Centre for Diabetes Complications	Australia	7.7	Wang, B., et al. miR-200a Prevents Renal Fibrogenesis Through Repression of TGF-beta2 Expression. Diabetes 60, 280-287 (2011).
237	26.3	2	4	6	Beckman Research Institute	USA	9.3	Putta, S., et al. Inhibiting MicroRNA-192 Ameliorates Renal Fibrosis in DiabeticNephropathy. Journal of the American Society of Nephrology 23, 458–469 (2012).
237	16.9	1	7	11	University of Groningen	Netherlands	9.3	Eijkelkamp, W.B.A., et al. Albuminuria is a target for renoprotective therapy independentfrom blood pressure in patients with type 2 diabetic nephropathy: Post hoc analysis fromthe Reduction of Endpoints in NIDDM with the Angiotensin II Antagonist Losartan(RENAAL) trial. Journal of the American Society of Nephrology 18, 1540-1546 (2007).
237	39.5	1	15	15	The University of Chicago	USA	45.5	Bakris, G.L., et al. Effect of Finerenone on Albuminuria in Patients With DiabeticNephropathy A Randomized Clinical Trial. Jama-Journal of the American MedicalAssociation 314, 884–894 (2015).
236	18.2	4	3	10	Baker Medical Research Institute	Australia	7.7	Thallas-Bonke, V., et al. Inhibition of NADPH oxidase prevents advanced glycation endproduct-mediated damage in diabetic nephropathy through a protein kinase C-alphadependentpathway. Diabetes 57, 460-469 (2008).
236	19.7	3	3	5	University of Texas	USA	9.3	Mehdi UF, Adams-Huet B, Raskin P, et al. Addition of angiotensin receptor blockade or mineralocorticoid antagonism to maximal angiotensin-converting enzyme inhibition in diabetic nephropathy. J Am Soc Nephrol. 2009;20:2641–2650.
236	13.1	NA	4	3	Department of Veterans Affairs Puget Sound Health Care System	USA	16.0	Young BA, Maynard C, Boyko EJ. Racial differences in diabetic nephropathy, cardiovascular disease, and mortality in a national population of veterans. Diabetes Care. 2003;26:2392–2399.
235	33.6	7	5	9	University of California San Diego	USA	3.1	Vallon, V., et al. SGLT2 inhibitor empagliflozin reduces renal growth and albuminuria inproportion to hyperglycemia and prevents glomerular hyperfiltration in diabetic Akitamice. American Journal of Physiology-Renal Physiology 306, F194-F204 (2014).
233	23.3	1	1	2	University of Madras	India	4.4	Palsamy P, Subramanian S. Resveratrol protects diabetic kidney by attenuating hyperglycemia-mediated oxidative stress and renal inflammatory cytokines via Nrf2-Keap1 signaling. Biochim Biophys Acta Mol Basis Dis. 2011;1812:719–731.
233	15.5	4	2	8	Vanderbilt University	USA	9.3	Zhao HJ, et al. Endothelial nitric oxide synthase deficiency produces accelerated nephropathy in diabetic mice. J Am Soc Nephrol. 2006;17:2664–2669.
232	17.8	1	6	14	Novartis Institutes for Biomedical Research	USA	7.7	Feldman DL, et al. Effects of aliskiren on blood pressure, albuminuria, and (pro)renin receptor expression in diabetic TG(mRen-2)27 rats. Hypertension. 2008;52:130–136.
229	12.7	3	1	10	Okayama University	Japan	7.7	Okada S, et al. Intercellular adhesion molecule-1-deficient mice are resistant against renal injury after induction of diabetes. Diabetes. 2003;52:2586–2593.
229	10.9	NA	3	7	Misato Junshin Hospital	Japan	4.5	Nakamura T, et al. Urinary excretion of podocytes in patients with diabetic nephropathy. Nephrol Dial Transplant. 2000;15:1379–1383.
226	14.1	6	3	7	University of Tokyo	Japan	8.9	Asaba K, et al. Effects of NADPH oxidase inhibitor in diabetic nephropathy. Kidney Int. 2005;67:1890–1898.
224	22.4	2	2	4	Kanazawa Medical University	Japan	7.7	Kitada M, Kume S, Imaizumi N, et al. Resveratrol improves oxidative stress and protects against diabetic nephropathy through normalization of Mn-SOD dysfunction in AMPK/SIRT1-independent pathway. Diabetes. 2011;60:634–643.
223	27.9	14	5	9	The Chinese University of Hong Kong	China	7.5	Zhong X, et al. miR-21 is a key therapeutic target for renal injury in a mouse model of type 2 diabetes. Diabetologia. 2013;56:663–674.
223	11.7	3	2	11	University of Melbourne	Australia	7.7	Forbes, J.M., et al. Reduction of the accumulation of advanced glycation end products byACE inhibition in experimental diabetic nephropathy. Diabetes 51, 3274–3282 (2002).
222	11.7	1	2	5	Soon Chun Hyang University	South Korea	9.3	Ha HJ, Yu MR, Choi YJ, et al. Role of high glucose-induced nuclear factor-kappa B activation in monocyte chemoattractant protein-1 expression by mesangial cells. J Am Soc Nephrol. 2002;13:9.
221	22.1	2	1	13	Okayama University	Japan	7.5	Kodera, R., et al. Glucagon-like peptide-1 receptor agonist ameliorates renal injurythrough its anti-inflammatory action without lowering blood glucose level in a rat modelof type 1 diabetes. Diabetologia 54, 965–978 (2011).
221	12.3	15	3	5	Biomedical Center	Sweden	7.5	Palm F, Cederberg J, Hansell P, et al. Reactive oxygen species cause diabetes-induced decrease in renal oxygen tension. Diabetologia. 2003;46:1153–1160.
220	14.7	5	6	11	Keio University	Japan	9.3	Ichihara, A., et al. Prorenin receptor blockade inhibits development of glomerulosclerosisin diabetic angiotensin II type 1a receptor-deficient mice. Journal of the American Societyof Nephrology 17, 1950–1961 (2006).

^a^ The number of total citations in the Web of Science Core Collection updated to December 2020.

^b^ The average citations adjusted by publication year.

^c^ The number of grants/funds received.

^d^ The number of institutes involved.

^e^ The number of authors.

^f^ The country of the first author.

^g^ The institute of the first author.

^h^ Journal impact factor (Journal Citation Reports™ 2020) where the article was published.

^i^ NA: not available.

### 3.2. Top cited articles per year

**Figure 1.**
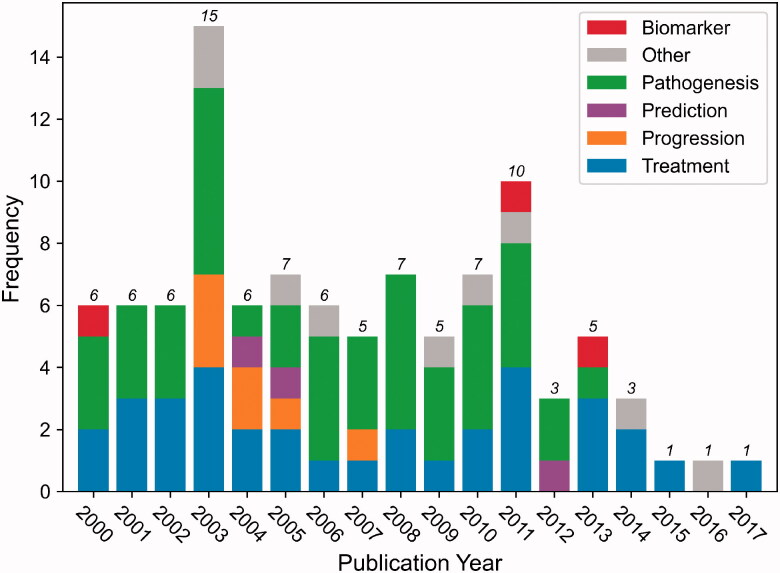
The publication year distribution of the 100 top-cited articles.

Publishing date spanned from 2000 to 2017, with a mean of 5.6 papers published per year. The largest number of literatures was published in the year 2003 (*n* = 15). The year 2003 and 2011 contributed 25 out of the 100 top-cited articles. Many top-cited articles focused on the pathogenesis of DKD (*n* = 44), followed by DKD treatment (*n* = 34) (Figure 1).

### 3.3. Journal distribution of top cited articles

**Figure 2.**
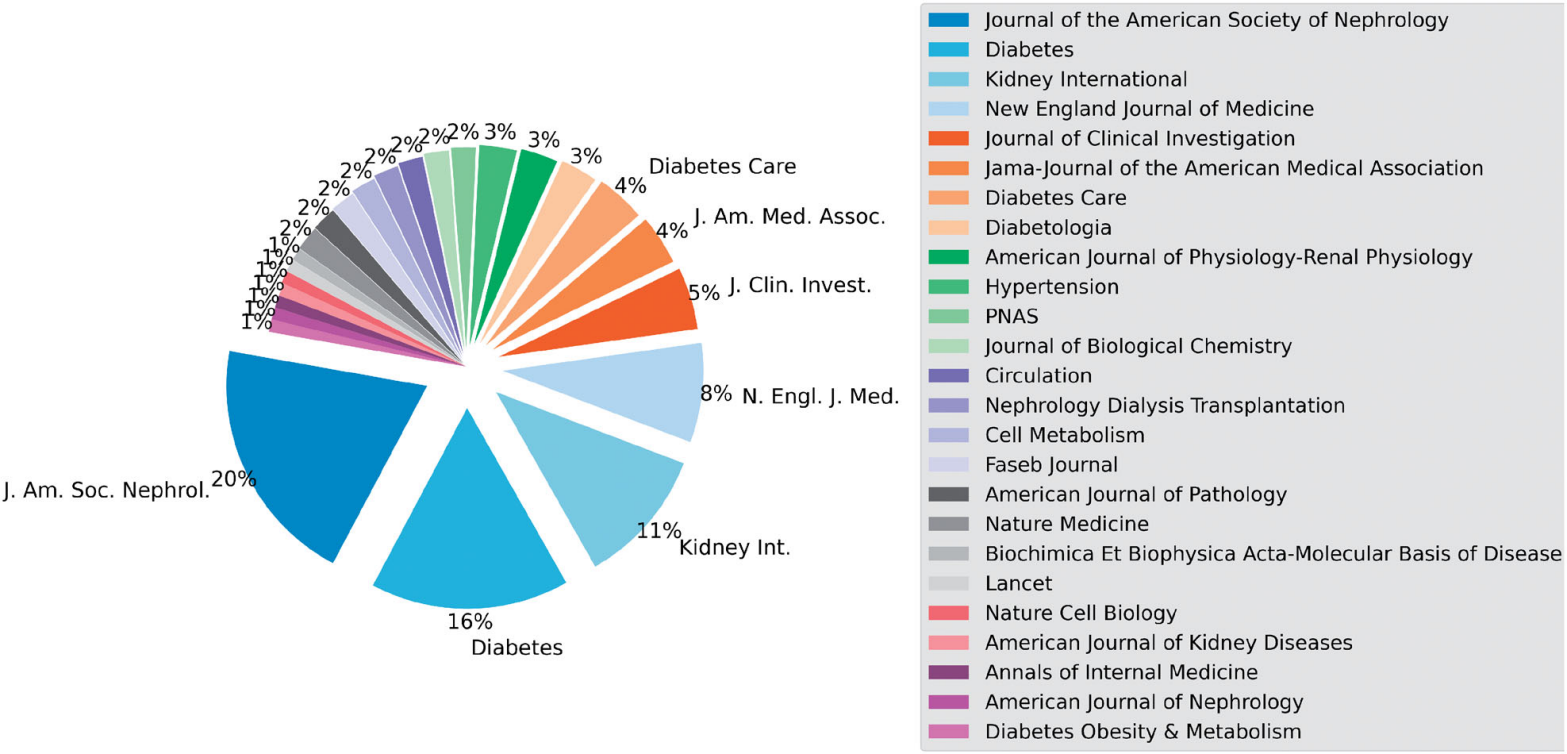
The journal distribution of the 100 top-cited articles.

The 100 top-cited articles were published in 25 different journals and the *Journal of the American Society of Nephrology* published the greatest number of these articles (20%), followed by *Diabetes* (16%), *Kidney International* (11%), and *New England Journal of Medicine* (8%) (Figure 2). The median IF of these journals was 9.27 (IQR, 7.72–12.9). The journal with the highest IF (74.7) was the *New England Journal of Medicine*. The journal *American Journal of Physiology-Renal Physiology* had the minimum IF (3.14) and had published three articles. Thirteen out of the 100 top-cited articles were published in seven different journals with an IF lower than 5.

### 3.4. Analysis of country and institute

**Figure 3.**
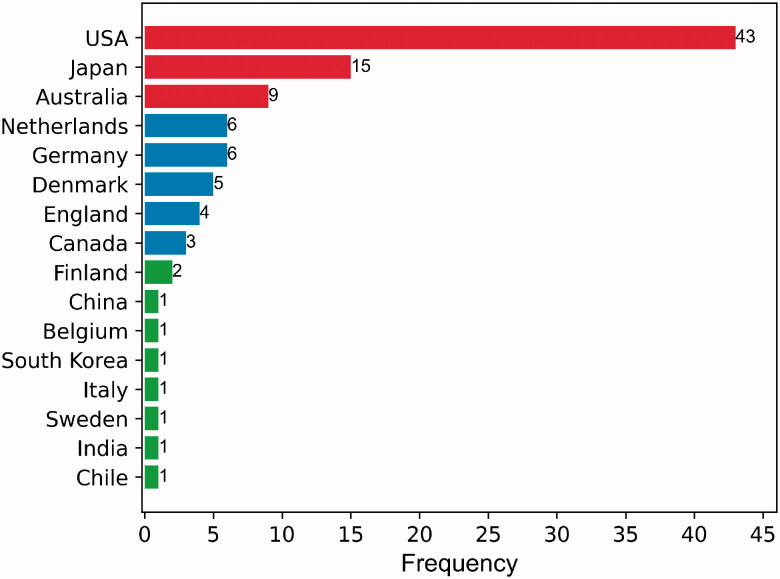
The country distribution of the 100 top-cited articles.

**Figure 4.**
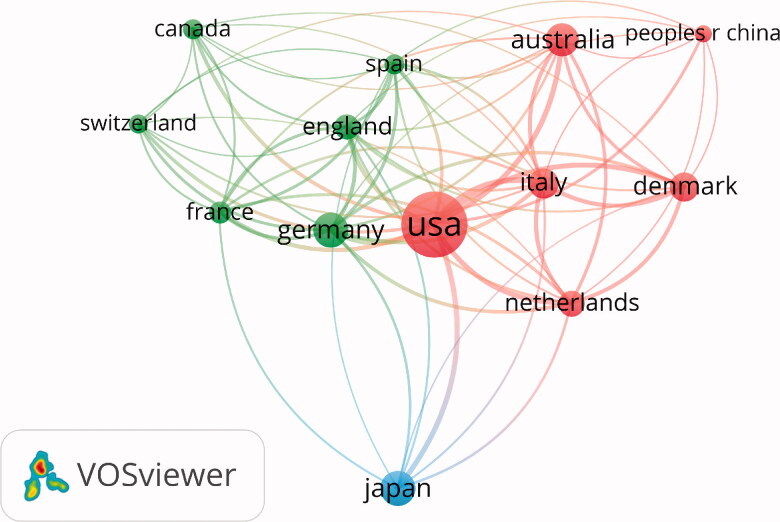
The network map of countries which coauthored five or more articles.

The first author of the publication determines the origin of the country and institution. The 100 most frequently cited articles emanated from 16 different countries. The United States contributed nearly half of these articles (*n* = 43), followed by Japan (*n* = 15) and Australia (*n* = 9) (Figure 3). Country coauthorship analysis reflects the collaborations between countries as well as the influence of countries in the field. Figure 4 shows the countries which coauthored five or more articles. The circle size is positively correlated with the number of articles coauthored by a country. The distance between two countries approximately indicates the relatedness of the countries in terms of coauthorship links. The United States was the most influential country, followed by Japan, Germany, and Australia.

**Figure 5.**
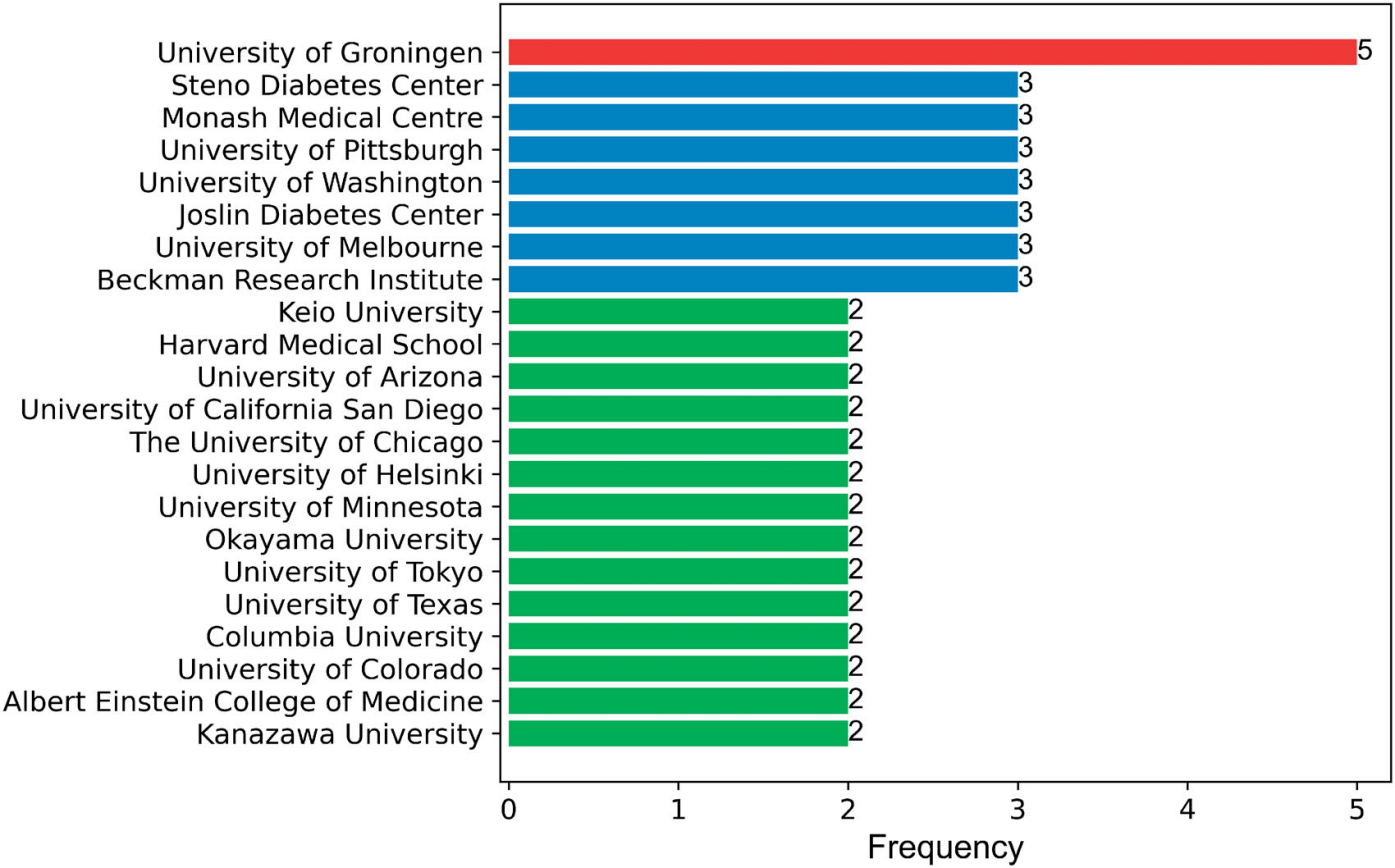
The institutions of two or more articles in the 100 top-cited articles.

**Figure 6.**
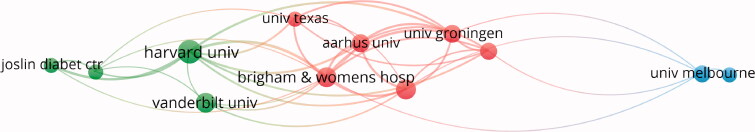
The network map of institutions which coauthored five or more articles.

The leading institutions with two or more top-cited articles are shown in Figure 5. The University of Groningen from the Netherlands was the leading institute contributing the highest number of papers (*n* = 5). University of Pittsburgh, Monash Medical Centre, Steno Diabetes Center, Joslin Diabetes Center, University of Washington, Beckman Research Institute, and University of Melbourne, each contributed three papers. An institution coauthorship network was plotted to visualize the collaborations between institutions as well as the influence of institutions in the field. Institutions that coauthored five or more articles are shown in Figure 6. Harvard University from the United States, the Steno Diabetes Center from Denmark, and the Brigham & Women's Hospital from the United States were the top three most influential intuitions with the largest number of coauthorship publications.

### 3.5. Analysis of authors

A total of 90 distinct first authors contributed to the 100 top-cited articles. The most productive author was de Zeeuw (*n* = 4), followed by Parving (*n* = 3). Five authors each contributed two articles (Table 2). The coauthors contributing five or more articles of the 100 top-cited articles were also summarized in Table 2 [7,23–48,50,51]. The median number of contributing authors for the 100 top-cited articles was nine (IQR, 6–12). A multicenter observational study (EDIC study) published in the year 2003 had 264 authors in collaboration.

**Table 2.** The authors of two or more articles as first author or of five or more as coauthor.

	Frequency	
Author name	As first author	As coauthor
de Zeeuw	4 [26,28,31,32]^a^	3 [23,35,46]
Parving	3 [7,24,37]	7 [23,26,28,31,32,43,46]
Steffes	2 [25,38]	0
Kato	2 [29,33]	1 [45]
Mann	2 [12,39]	0
Perkins	2 [30,41]	1 [27]
Ichihara	2 [34,51]	0
Cooper	0	10 [23,28,36,40,42,44,46–48,50]
Remuzzi	0	8 [23,26,28,31,32,37,46,47]
Shahinfar	0	5 [23,26,28,35,46]

^a^ The sequence number of corresponding references.

### 3.6. Analysis of correlations

There was no correlation between average citations and the number of authors (*r* = 0.10, *p*>.05), the number of institutes (*r* = 0.30, *p*<.05), or the number of funds (*r* = 0.04, *p*>.05), respectively.

### 3.7. Analysis of type of study

Among 100 top-cited articles, 30 publications were experimental animal study, followed by observational study (*n* = 24), randomized controlled trial (RCT) (*n* = 21), and *in vivo* (animal) and *in vitro* study (*n* = 20). Half of the top-cited papers involved diabetic animal models (*n* = 50) (Table 3).

**Table 3.** Classification of the 100 top-cited articles based on type of study.

Type of Study	Frequency
*In vivo* study (animal)	30
Observational study^a^	24
Randomized controlled trial^a^	21
*In vivo* (animal) and *in vitro* study	20
*In vitro* study	3
Single arm study^a^	2

^a^ Subtype of clinical study involved in human subjects.

### 3.8. Analysis of keywords

As the author keywords of some top-cited articles are not available, all keywords (including both Author keywords and Keywords Plus) were used for keywords analysis. A total of 577 character-different keywords were identified from the 100 top-cited articles. After manually confirmed and refined, the top 10 most frequent keywords and their frequency are summarized in Table 4.

**Table 4.** The top 10 most frequent keywords of the top-cited articles.

Rank	Keywords	Frequency
1	Renal-disease	42
2	TGF-beta^a^	24
3	Diabetic nephropathy	21
4	Oxidative stress	20
5	Diabetes mellitus	19
6	Proteinuria	17
7	Converting enzyme-inhibition	13
8	Albuminuria	13
9	Angiotensin-ii	12
10	Hypertension	12
11	Progression	12

^a^ Transforming growth factor-β.

## 4. Discussion

Bibliometrics is a method to quantitatively analyze research publications and their performance [3,52]. DKD is a global health epidemic with an explosive expansion of related scientific publications during the past few decades. However, bibliometric analysis of top-cited articles in this field has yet to be performed. This bibliometric study identified the 100 top-cited articles in the field of DKD and evaluated their main characteristics quantitatively. The analysis explored the distribution of these articles in terms of citation ranking, publication year, publication journal, country and institution of origin, authorship, and keywords. In addition, we created the network maps to analyze country coauthorship and institution coauthorship.

The most-cited article was by Brenner published in 2001 (cited 4753 times) [23]. Notably, the top two most-cited articles based on total citations are all RCTs related to RAAS interruption (the investigational products are losartan and irbesartan, respectively) [23,24], and this is in accord with the important role of ACEI or ARB in the clinical management of DKD. The analysis of the 100 top-cited articles reveals the bias of time on total citations. Most of these articles (*n* = 69) were published a decade ago and none in recent 5 years appears in the list of top 15. Thus, we rearranged these articles according to average citations per year. Consequently, a RCT of liraglutide (a GLP-1RAs) published in 2017 ranked 3rd, while in the total citations it only ranked 25th [12]. Similarly, two articles related to SGLT2 inhibitor published in 2013 and 2014 ranked much higher based on average citation per year [9,27]. Despite a result of increased cardiovascular events, a clinical trial of bardoxolone methyl ranked 6th, indicating anti-inflammatory agents received much attention in DKD [31]. Typically, citation of a scientific publication began substantially one or two years after publication, reached a maximum after 3–5 years, and then decreased to a lower level. A publication may be ignored initially, and several decades may need to accurately assess the performance of a scientific work [4]. Thus, represented by the four articles above, newly published articles that have high average citations per year may reflect the emerging trends in the field of DKD and accrue more citations over time.

As shown in Figure 1, nearly three-quarter of articles involved pathogenesis and treatment, indicating that nephrologists are diligently exploring new pathogenesis, key pathways, therapeutic targets, and promising drugs to prevent, arrest, treat, and reverse DKD [53]. However, more influential papers are needed to investigate the prediction models and valuable biomarkers of DKD.

A high proportion of top-cited articles were published in the *Journal of the American Society of Nephrology* (IF: 9.274), which is one of the leading specialist journals publishing both basic and clinical research relevant to a broad discipline of nephrology. Although most top-cited articles were published in journals with high IF (the lower quartile of the IF was 7.72), 13 articles were published in seven different journals with an IF lower than 5. This demonstrates an inconsistency between the publishing journal IF and the influence of specific articles as well as the potential limitation of IF in predicting influential papers [54]. The IF is a journal-level metric to evaluate the impact of the academic journal and is largely influenced by journal publication policies and varies across time [55].

Nearly half of the 100 top-cited articles originated from the United States. The United States was also at the center of the country coauthorship network map, reflecting a close collaboration with many other countries, such as Germany, Italy, and the Netherlands. This indicates the leading academic position of the United States in the field of DKD. Notably, Japan published the second-highest number of top-cited articles. However, Japan had a relatively weak collaborative link with other countries in DKD research, reflected by a long distance from other countries in the country coauthorship network map. Oppositely, although no or few articles originated from Spain and Italy, they were actively involved in the collaboration with other countries. The map shows a close collaborative link between developed countries, such as the United States and many European countries. Considering major scientific research projects such as multi-center clinical study are conducted primarily through a network of regional or global collaboration, Asian countries such as Japan, China, and South Korea should deepen their cooperative relationship in research frontiers with the United States and European countries.

Among 22 leading institutes contributing two or more articles, 13 institutes were from the United States which may be attributed to generous funding support, well-trained researchers with long working hours, scientific creativity, and mobility of researchers across countries [56]. The University of Groningen from the Netherlands contributed five top-cited articles, and many of these studies were clinical trials focusing on the albuminuria. However, in the institution coauthorship network map, Harvard University from the United States contributed the largest number of coauthorship publications. Notably, although Brigham & Women’s Hospital and Aarhus University contributed few top-cited articles as the first affiliation, they had a strong collaborative link with other leading institutions. These results suggest the United States and many European countries are the leaders in the frontiers of DKD research and are well cooperated to yield high-quality papers.

De Zeeuw and Parving were top researchers in the field of DKD. Notably, represented by Cooper, Remuzzi, and Shahinfar, these authors were not listed as the first author of any of the top-cited articles, but they were actively involved in the academic collaboration, and are important research specialist in this filed. Coauthorship analysis gives a new insight into the contribution of dedicated scholars, even if they are not listed as the first author in any top-cited paper [57]. The majority of the top-cited articles were completed by six or more authors (the lower quartile of the number of coauthors was 6), reflecting a trend of more cooperation in high-quality papers. Notably, many clinical studies were conducted by hundreds of sub-investigators, who were list as ‘Group Authors’ in the published article, and their contribution should be gratefully acknowledged. The average citations per year primarily depends on the content, quality, novelty, and publishing journal of the article, thus it is understandable that the average citations per year was uncorrelated with the number of authors, the number of institutes, or the number of funds.

Diabetic animal models were involved in half of the top-cited articles, indicating its important role in the basic research of DKD. Multiple rodent models, such as streptozotocin-induced diabetic model, db/db diabetic mice, are widely used to experimentally simulate the pathophysiological mechanism of human diabetes mellitus and its complications. However, the degree of fidelity in replicating the main features of human DKD and the diversity in human diabetic patients are still tricky issues to be solved [58,59]. Accordingly, clinical study is another critical aspect of DKD research. Observational clinical study ranked 2rd in the top-cited list, which plays a unique role in DKD research as a well-designed prospective observational study enables a long-term follow-up (e.g., decade) of DKD patients in real world [60]. DKD patients are frequently concomitated with other diseases, and this part of patients may fail to enter the randomized control trial due to relative strict inclusion and exclusion criteria. Thus, results from some RCTs may fail to represent the overall characteristics of the DKD population. Nevertheless, large multi-center randomized control trial is the golden standard of assessing therapeutic efficiency and is considered as one of the highest levels of evidence in evidence-based medicine [61]. The renal benefits of classic ACE inhibitor or ARB, as well as newly emerging SGLT2 inhibitor in DKD patients, are all demonstrated by large-scale RCTs and then recommended in authoritative guidelines [23,24,62–64]. In a word, the basic study on animal or cell level and clinical study on human subjects are two mutually complementary aspects in DKD research.

The analysis of keywords provides insight into the classic hotspots as well as emerging trends. As expected, renal-disease, diabetic nephropathy, and diabetes mellitus were frequent keywords in these top-cited articles. Transforming growth factor-β (TGF-β) ranked 2rd in the frequency list, which is a classic molecular receiving much attention in the field of DKD since the 1990s. TGF-β induces fibrotic and inflammatory genes expression in the diabetic kidney. However, how exactly hyperglycemia induces TGF-β increasing remains to be elucidated [65,66]. The fourth most frequently used keyword was ‘oxidative stress’, which is involved in the initiation and progression of DKD [67]. The keywords ‘Converting enzyme-inhibition’ and ‘Angiotensin-ii’ indicated RASS interruption is a research hotspot in the field of DKD. Proteinuria and albuminuria are key clinical signs of DKD and are involved in glomerulosclerosis. Currently, as DKD is still an irreversible condition, it is not surprising that the keyword ‘Progression’ was highlighted in many top-cited articles. Hypertension, as an important risk factor of DKD, is also an intrinsic characteristic of DKD. A proper blood pressure-lowering strategy is essential for preventing the onset, progression, and cardiovascular events of DKD [68,69]. Notably, the keywords frequency of the 100 top-cited articles is not appropriate for predicting emerging new trends due to the bias of publication time on the number of citations. However, keywords such as ‘SGLT2 inhibitor’ and ‘GLP-1RAs’, were burst in newly published top-cited articles [9,12, 27,49]. Although there is a low frequency of occurrence among the 100 top-cited articles, these keywords imply the emerging trends in the field of DKD.

Zou et al. [70] conducted a bibliometric analysis to analyze 27,577 publications of DKD from 2000 to 2017 and concluded that Parving, Harvard University, and USA were the most influential scholar, institute, and country in this field, respectively. The above results are consistent with our analysis and indicate that the top 100 list of articles represents the overall scientific outputs well. The classic research hotspots identified by Zou et al. are similar to our result but with more details. However, differing from Zou et al., our analysis shows that ‘SGLT2 inhibitors’, ‘GLP-1Ras’, and anti-inflammatory agents are the emerging trends of DKD and reveals an insufficiency of high-quality researches on the prediction and biomarker of DKD.

There are several limitations in our study. First, the 100 top-cited articles were identified according to the total citation number, which is inherently affected by publication year. Newly published influential papers are more likely to be eliminated. Second, the analysis failed to reveal some promising research directions as the articles related to them acquired insufficient citations yet, such as mineralocorticoid receptor antagonists, endothelin receptor A inhibition, autophagy activators, and epigenetic remodeling [47,71]. Third, as a methodology, citation analysis has some potential biases, such as self-citation bias, in-house bias, journal bias, and English language bias [72]. Fourth, Thomson Reuters Web of Science, a large-scale global citation database, was used to retrieve the top-cited articles. However, the combination of Web of Science with other databases, such as Scopus and Google scholar, would enable a more robust bibliometric analysis.

## 5. Conclusions

This analysis provides a historical perspective on the 100 top-cited articles in the field of DKD and helps in identifying the milestones, inadequacies, classic hotspots, and emerging trends. The United States, Japan, and many European countries are academic leaders with discovery, innovation, productivity, and collaboration. Pathogenesis and treatment are core themes in DKD research, however, high-quality researches on the prediction and biomarkers are insufficient. Basic studies have an advantage in exploring molecular mechanisms, while clinical studies stand out in assessing the therapeutic efficiency of interventions. TGF-β, oxidative stress, proteinuria, and RAAS interruption are classic research topics, while SGLT2 inhibitors, GLP-1 receptor agonists, and anti-inflammatory agents represent the recent research hotspots. New analyzing methodologies and metrics are needed to deeply assess the actual impact of these top-cited articles on clinical practice.

## Abbreviations

ACEI: angiotensin-converting enzyme inhibitor
ARB: angiotensin receptor blockade
DKD: diabetic kidney disease
ESRD: end-stage renal disease
GLP-1: glucagon-like peptide 1
GLP-1RAs: glucagon-like peptide 1 receptor agonists
IF: impact factor
IQR: interquartile ranges
RAAS: renin–angiotensin–aldosterone system
RCTs: randomized controlled trials
SDs: standard deviations
SGLT2: sodium-glucose cotransporter 2
TGF-β: transforming growth factor-β
